# Development of *Health Enhancement Lifestyle Profile - Taiwanese Short Form Version (HELP-T-SF)* for the community-dwelling elderly

**DOI:** 10.1371/journal.pone.0336695

**Published:** 2025-11-12

**Authors:** Pei-Chi Su, Hui-Fen Mao, Wen-Chen Cheng

**Affiliations:** 1 School of Occupational Therapy, College of Medicine, National Taiwan University, Taipei, Taiwan; 2 Department of Gerontological and Long-Term Care Business, College of Nursing, Fooyin University, Kaohsiung, Taiwan; 3 Professional Education Center of Holistic Care in Aging, Fooyin University, Kaohsiung, Taiwan; 4 Department of Physical Medicine and Rehabilitation, National Taiwan University Hospital, Taipei, Taiwan; 5 Health Science and Wellness Research Center, National Taiwan University, Taipei, Taiwan; Taipei Medical University, TAIWAN

## Abstract

**Background:**

The Health Enhancement Lifestyle Profile - Taiwan Version (HELP-T) assesses the lifestyle profiles of the older adults through participation in activities across seven domains: exercise, diet, social and productive activities, leisure, activities of daily living, stress management and spiritual participation, and other health behaviors. This study aimed to develop a short form of HELP-T (HELP-T-SF) to reduce assessment time and evaluate its psychometric properties.

**Methods:**

This three-phase study comprised item reduction using archival data (2012–2013), field testing (n = 223; 2023), and psychometric evaluation (n = 117; 2024) among community-dwelling older adults. Data collection included the HELP-T-SF, original HELP-T, WHO-5 Well-Being Index, and quality-of-life questions. Analysis employed classical test theory.

**Results:**

The finalized HELP-T-SF, consisting of 20 items. Internal consistency for the total score was Cronbach’s α = 0.78 (95% CI: 0.54 to 0.89); test–retest reliability over 7–14 days was ICC (3,1) = 0.78 (95% CI: 0.54 to 0.89); correlation with the long form was r = 0.75 (95% CI: 0.56 to 0.86). Convergent validity showed moderate correlations with well-being and quality of life. The short form reduced assessment time to 10–15 minutes.

**Conclusions:**

The HELP-T-SF is a valid tool for assessing lifestyle profiles in community-dwelling older adults, assisting practitioners in lifestyle medicine for understanding older adults’s lifestyle profile, setting client-centered goals and designing personalized lifestyle interventions.

## Introduction

According to the World Population Prospects 2022, the global population aged 65 and older is growing more rapidly than younger age groups, projected to rise from 10% in 2022 to 16% by 2050 [1] In Taiwan, this demographic is expected to reach 4.70 million by 2025, comprising 20.1% of the national population [2]. Chronic diseases pose a major global health challenge. Addressing modifiable lifestyle behaviors and non-medical determinants is essential for effective prevention and management [3], underscoring the value of lifestyle-based interventions [4,5]. The healthy aging framework of World Health Organization (WHO) and large epidemiological studies confirm that lifestyle is a key driver of functional ability and well-being in old age [6,7] Therefore, Effective chronic disease management and health promotion can significantly reduce disability and dementia rates among older adults, highlighting the importance of lifestyle interventions.

“Lifestyle” is a complex, multi-dimensional concept that encompasses various activities, whether routinely or intermittently, over a prolonged period. It aggregates of habitual behaviors and choices that directly influence health, functional ability, and well-being in older adults [6,8–10]. The WHO healthy lifestyle framework specifically operationalizes lifestyle within the paradigm of “functional ability,” emphasizing the capacity to be and do what one values, shaped by both intrinsic capacity and environmental factors [6]. This framework integrates domains including physical activity, diet, smoking, alcohol use, sleep, stress management, and social participation, and recognizes the impact of social determinants and the built environment on these behaviors [6,8]. Lifestyle Medicine (LM), an emerging evidence-based field that focuses on preventing, managing chronic diseases through lifestyle modifications. It is grounded in preventive medicine. According to Sagner et al. (2014), LM is “a branch of evidence-based medicine in which comprehensive lifestyle changes are employed to prevent, treat, and reverse the progression of chronic diseases by addressing their underlying causes [11]”. Nowadays, LM addresses the root causes of chronic diseases through six key pillars: 1) a whole-food, plant-predominant diet, 2) physical activity, 3) restorative sleep, 4) stress management, 5) avoidance of risky substances, and 6) positive social connections [12]. Occupational science proposes a broadened conceptualization of lifestyle, viewing it as a pattern of daily occupations and routines that support or hinder health, emphasizing the meaning, context, and adaptability of activities in later life. This perspective highlights the importance of engagement in meaningful activities, autonomy, and the interplay between personal capabilities and environmental supports [8,10]. The *Occupational Therapy Practice Framework: Domain and Process—Fourth Edition (OTPF, 4th)* provides a comprehensive approach to participation in meaningful activities, aligning with the WHO’s definition of health [13]. OTPF emphasizes “supporting health and participation in life through engagement in occupation” [14] and identifies seven domains: 1) exercise, 2) diet, 3) social and productive activities, 4) leisure, 5) activities of daily living (ADLs) (including sleep), 6) stress management and spiritual participation, and 7) other health promotion and risk behaviors [15]. This occupation-based model aligns closely with the WHO’s concept of healthy aging, which advocates for functional ability through lifestyle optimization in physical, mental, and social domains. In this way, the OTPF-based perspective complements the predominantly biomedical focus of the Lifestyle Medicine model [16], offering a holistic lens that incorporates subjective experience, environmental fit, and behavior sustainability.

Under those conceptual foundation above, older adults’ lifestyles differ significantly from younger populations. Compared to younger people, older adults after retirement may spend more time for leisure activities, which constitute a major occupational area [17]. Engagement in leisure activities that support physical and cognitive function is linked to increased longevity and improved mental well-being [18]. Leisure participation has also been associated with reduced depression and enhanced quality of life [19]. Therefore, understanding and addressing lifestyle patterns in older adults is essential. Assessing changes in lifestyle engagement can help identify individual needs and promote a balanced life.

A well-designed lifestyle assessment tool for older adults is crucial for evaluating health-related behaviors and living conditions. Lifestyle factors are major, modifiable determinants of health outcomes, functional independence, and quality of life in this population. It may identify areas for improvement in health knowledge and behaviors forming the foundation for assessing and monitoring outcomes for clinical practitioners to integrate LM principles with the OTPF framework, supporting individuals in adopting comprehensive health-promoting activities [20]. Many existing evidence demonstrates that healthier lifestyle profiles in older adults are associated with slower declines in physical, psychological, cognitive, and social functioning, as well as reduced risk of disability and chronic disease. For example, longitudinal studies show that healthy lifestyle behaviors predict better maintenance of gait speed, cognitive function, mood, and social engagement over time, and are linked to successful aging and independence in activities of daily living [21–23]. LM begins with an assessment of several dimensions relevant to disease prevention, which subsequently guides lifestyle interventions. However, it is medical model based and not specifically designed for older adults. Thus, a comprehensive lifestyle assessment targeting health-promoting activities in older adults is vital for identifying at-risk individuals, optimizing health, preventing decline, implementing effective lifestyle interventions, and monitoring progress [21–25].

The Health Enhancement Lifestyle Profile – Taiwanese Version (HELP-T) provides a structured approach to assessing health-promoting activities and behaviors in older adults, which rooted in the OTPF. HELP-T developed from the HELP (originally from the United States) by translated into Traditional Chinese with cultural modifications in Taiwan in 2012. A Japanese version (HELP-J) has also been developed later in 2024 [26]. HELP and HELP-T are self-reported tools evaluating activity frequency over the past three months. The HELP consists of 56 items. It uses a scale ranging from 0 to 5, categorized as: 1) never, 2) 1–2 days per month, 3) 1–2 days per week, 4) 3–4 days per week, 5) 5–6 days per week, and 6) 7 days per week. Psychometric properties were evaluated using both Classical Test Theory (CTT) and Item Response Theory (IRT), demonstrating satisfactory results of reliability and validity [15,27,28]. The HELP-T includes three culturally relevant items and demonstrates strong reliability (internal consistency = 0.82; test-retest reliability ICC = 0.92) and validity (correlations between subscale and total scores, Spearman’s rho = 0.41–0.67, p < 0.01). However, due to the cognitive load during responding, the 6-point scale was later simplified to a 3-point scale [29].

Despite its utility in community settings, HELP-T’s completion time (20–40 minutes) limits its practicality. In addition, older adults reported difficulty recalling the frequency of participating in a specific activity or behavior over the past three months. Therefore, the recall period was modified to the past month. To enhance accessibility and application in community practice, this study aims to develop a shortened version of HELP-T for efficient assessment of healthy lifestyle engagement among older adults. The reliability and validity of the short form HELP-T, as well as its correlation with the long version will be further examined.

## Methods

### Study design

**Fig 1:**
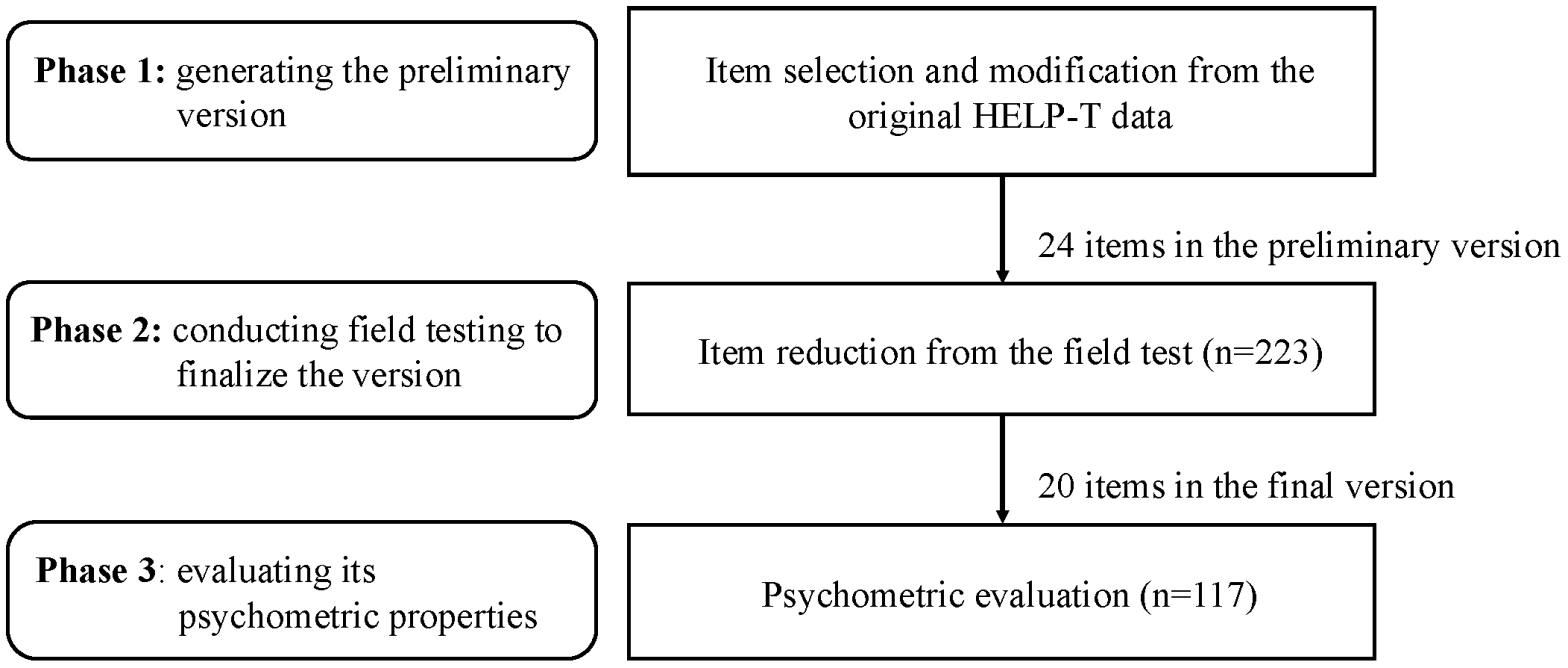
Phases of the HELP-T-SF study.

The current study aimed to develop and validate the short form HELP-T (HELP-T-SF) through three phases: 1) generating the preliminary version, 2) conducting field testing to finalize the version, and 3) evaluating its psychometric properties (Fig 1). The study was approved by the Institutional Review Board of National Taiwan University Hospital (201203041RIC and 202308040RINA). Data of Phase 1 were collected from 17/04/2012 to 16/04/2013, and Phase 2–3 were collected from 12/09/2023 to 11/09/2024. All participants have completed and signed the consent forms.

### Phase 1: Generating the preliminary version of the HELP-T-SF

In Phase 1, we analyzed an initial dataset collected in 2012–2013 from 372 community-dwelling older adults using a 6-point Likert scale. This version, applied exclusively for item selection and reduction, provided greater response granularity and enabled more precise psychometric analyses to identify representative items. The 3-point version of the HELP-T, later published in the 2018 PLOS ONE article, was derived by collapsing categories from the original 6-point scale, with high correlation between the two formats (r = 0.985). The simplified 3-point scale enhanced ease of response and consistency in practical settings. Item selection was based on multiple criteria, including central tendency (mean, median), correlations with subscales and total scores, internal consistency, discrimination between high- and low-scoring groups, and gender differences.

Following this analysis, the research team engaged in discussions to refine the content of the preliminary version, including item selection, item combination and item rewriting. Our goal was to shorten the HELP-T while effectively measuring the “lifestyle profile” as a concept. This led to the consolidation of several subscales (including exercise, diet, social and productive activities, leisure, ADLs, stress management and spiritual participation, and other health promotion and risk behaviors [15]) into one scale, ensuring that each subscale contained 2–4 items. Additionally, we noted that reverse-worded questions posed comprehension challenges and resulted in suboptimal statistical results. Consequently, direct questions were incorporated into the preliminary version of the HELP-T-SF to enhance clarity.

### Phase 2: Conducting field testing to finalize the version of HELP-T-SF

A cross-sectional study was conducted to finalize the HELP-T-SF.

#### Participants.

We recruited 223 community-dwelling older adults (aged > 55) who were cognitively intact and able to communicate in Mandarin or Taiwanese. Convenience sampling was employed to recruit community-dwelling older adults from partnering community settings throughout the southern region of Taiwan. Participants were invited to join the study if they were already engaged in the community setting. In a few cases, additional eligible individuals such as caregivers or family members who accompanied the participants also expressed interest and were included, provided they met the criteria.

#### Data collection.

Several well-trained on-site interviewers with relevant professional backgrounds conducted the data collection, which included the following data.

*Basic information.* Basic demographic information included age, gender, education level, marital status, living arrangement, chronic diseases, regular medical care or medications, and self-rated health status (4-point Likert-scale).

*Preliminary version of HELP-T-SF.* The version developed from Phase 1.

*HELP-T.* To examine the correlation between the HELP-T-SF and the original HELP-T, 30 participants were randomly selected to complete the full HELP-T. This assessment measures the frequency of engaging in health-promoting or health-related behaviors over the past three months, across seven subscales. The questionnaire consists of 59 items, with scoring ranging from 0 to 2 points (None or 1–3 days/month = 0, 1–3 days/week = 1, 4–7 days/week = 2) per item [29].

*The WHO-5 Well-Being Index*. *The WHO-5 Well-Being Index*. Developed by the WHO, this scale comprises 5 questions addressing an individual’s mental well-being. A higher score signifies greater sense of well-being. The total score is multiplied by 4 to provide a percentage score ranging from 0 to 100 [30].

*Quality of life.* From a pragmatic perspective, two items were selected from the WHOQOL-BREF questionnaire to reduce response burden [31,32]: “How would you rate your quality of life?” and “How satisfied are you with your health?” Each item offers 5 response options, ranging from “very poor” to “very good” [33].

The WHO-5 Well-Being Index, the Quality of life, self-perceived health, and health satisfaction were assessed to evaluate the convergent validity of the HELP-T-SF.

#### Item reduction.

According to the results of the data analysis, the final version of HELP-T-SF was determined based on six criteria: mean, standard deviation, distribution of the rating scale, item-total correlation (Pearson’s r), high-low group difference (Cohen’s d), and internal consistency (△Cronbach’s α if an item was deleted).

### Phase 3: Evaluating the psychometric properties of HELP-T-SF

A descriptive analysis of the demographic and health-related basic data of the participants was performed using percentages, frequencies, means, standard deviations, and medians. The reliability and validity of the HELP-T-SF were assessed using CTT with a subset sample size of n = 117 from Phase 2.

#### Reliability.

Cronbach’s alpha (α) was employed to assess the internal consistency of the total score for the HELP-T-SF. An alpha value of 0.70 or higher is typically considered acceptable [34]. We calculated the 95% confidence interval for α to provide precision around the estimate. A subgroup of 38 participants completed the HELP-T questionnaire twice, with an interval of 7–14 days to evaluate test-retest reliability. We estimated test–retest reliability using a two-way mixed-effects, absolute-agreement, single-measure intraclass correlation [ICC(3,1)] with 95% confidence intervals. Intra-class correlation coefficient (ICC) was utilized as a reliability measure for questionnaires, demonstrating the consistency of participant responses over time. An ICC value greater than 0.75 is generally considered high, while values between 0.75 and 0.40 are seen as moderate, and those below 0.40 are considered low [35].

In addition, Pearson correlation coefficients between the HELP-T-SF and the original HELP-T were reported to assess concurrent validity, with 95% CIs.

#### Validity.

The correlation between the HELP-T and the HELP-T-SF was assessed by comparing scores in a randomly selected group of 33 participants, aiming for a moderate to high correlation (r = 0.6 or above) between the two groups. To examine convergent validity, we analyzed the correlations between the HELP-T-SF behavior construct and four psychosocial indicators: mental well-being, self-perceived quality of life, self-perceived health, and health satisfaction. These constructs were selected based on prior evidence indicating their associations with health-related lifestyle behaviors among older adults. While conceptually related, they do not represent the same construct directly. Correlation coefficients below 0.29 were considered weak, between 0.30 and 0.49 as low, between 0.50 and 0.69 as moderate, and higher than 0.70 was considered strong correlation. The lower the correlation, the more it indicates that the measurements tool show different constructs [36]. In this study, we hypothesized the correlation was low to moderate, reflecting the related but distinct nature of these domains and lifestyle engagement.

SPSS 25 (IBM Corp., Somers, NY, USA) was utilized for all statistical analyses. Statistical significance was set at p < 0.05, and for multiple comparisons, adjustments were made using the Bonferroni correction.

## Results

### Phase 1: Generating the preliminary version of the HELP-T-SF

In Phase 1, a statistical item selection process and discussions within the research team led to the identification of 14 original items. Additionally, eight items were created by combining multiple original items, and two items were rewritten using positive phrasing to replace the reverse items (see Table 1).

**Table 1:** Preliminary version of the Health Enhancement Lifestyle Profile – Taiwanese Short Form Version (HELP-T-SF) and a summary of item selection.

			Statistical criteria						
Item		Note	not meet (n)	a. Mean	b. Median	c. Item–Subscale Correlation	d. Item–Total Correlation	e. Cronbach’s α Change (if item deleted)	f. High vs. Low Group Comparison
Pre01	Walk for 20 min	Original item	0						
Pre02	Do yoga or stretching exercise	Original item	0						
Pre03	Do moderate intensity physical activity for 30 minutes	Combined item	–						
Pre04	Three servings of healthy foods rich in protein	Original item	0						
Pre05	Three servings of fruits or vegetables	Original item	0						
Pre06*	Two servings of foods high in cholesterol, sodium or saturated/trans fat	Combined item	–						
Pre07	Family caregiving, volunteering, or paid work	Combined item	–						
Pre08	Participate in social gatherings	Combined item	–						
Pre09	Gathering with family	Original item	0						
Pre10	Contact with family members who don’t live together	Original item	0						
Pre11	Engage in hobbies or interest activities at home	Combined item	–						
Pre12	Engage in outdoor hobbies or interest activities	Combined item	–						
Pre13	Engage in cognitive activities	Combined item	–						
Pre14*	Stay up late at night	Original item	4	x	x		x		x
Pre15*	Not having enough rest in daytime	Original item	2				x		x
Pre16	Do family chores	Combined item	–						
Pre17	Have a sense of satisfaction in life	Original item	0						
Pre18	Do things that bring good moods	Original item	0						
Pre19	Do relax activities	Original item	0						
Pre20	Read health-related articles	Original item	0						
Pre21	Watch health-related programs	Original item	0						
Pre22	Monitor health at home	Original item	0						
Pre23	Have good quality of sleep	Positive phrasing of Pre14	–						
Pre24	Have enough rest in daytime	Positive phrasing of Pre15	–						
**Total 24 items**									

*Reverse item.

The following conditions do not meet the criteria:

a. Mean: Items with average scores ≤ 1 or ≥ 4.

b. Median: Medians of 0 and 5.

c. Correlation with Subscale: If the calculation was not significant or negatively significant.

d. Correlation with Total Score: insignificance or negatively significant coefficient between each item and the total score.

e. Internal Consistency Reliability Coefficient (with Subscale): The increasing change in reliability index “Cronbach’s α if item deleted” after removing the item for each of the seven subscales.

f. High-Low Group Differences: No significant differences between the high (top one-third of high scorer) and low score group (bottom one-third of low scorers).

#### Item selection.

From the total of 59 items in the HELP-T, 14 original items were selected based on specific criteria. An item was chosen if it had fewer than three discrepancies. If the cumulative number of items failing to meet this criterion reached three, they were considered underperforming and reviewed for potential removal or adjustment. One item remained, despite having four discrepancies, as it pertained to the topic of sleep.

#### Item combination.

Eight items were generated by consolidating several individual items into broader categories of activities. For instance, within the Leisure subscale, three items—hobbies or interest activities at home, outdoor hobbies or interest activities, and cognitive activities—were merged from a total of eight original items after selection. The original questions categorize each subscale of activity in very detail, such as “going out for sports events or movies” and “picnics, fishing, or sailing.” We should respect individuals’ choices of suitable interested activities, provided they engage to a certain extent within the same characteristic. Consequently, the items mentioned above were combined into the item “outdoor hobbies or interest activities.”

#### Item rewriting.

The research team added two items with positive phrasing: “Have good quality of sleep” as an alternative to “Stay up late at night,” and “Have enough rest during the day” as an alternative to “Not having enough rest during the day.”

Consequently, the preliminary HELP-T-SF consisted of 24 items, including 21 positively phrased items and 3 reverse items.

### Phase 2: Conducting field testing to finalize the version of HELP-T-SF

In Phase 2 of field testing, 223 older adults, with a mean (standard deviation) age of 71.42 (8.80) years (ranging from 55 to 98 years) participated; of these, 170 participants (76.2%) were women (Table 2). Based on the results of analysis, four items were identified for removal. Three of them were reverse-worded—one related to diet and two concerning daily life rest and sleep— being removed due to a difference between high- and low- scoring group (Cohen’s d), showing poor discrimination. Furthermore, their item-total correlations were nonsignificant, and the internal consistency was inadequate, indicated by an increase in Cronbach’s α when the items were deleted (see Table 3). In addition, one positively worded item, “Have enough rest during the day” was also removed due to its poor discrimination and difficulty in comprehension by respondents. The final version is comprised of 20 items with no reverse items, and each subscale contains 2–4 items. The scoring ranging from 0 to 2 points (None or 1–3 days/month = 0, 1–3 days/week = 1, 4–7 days/week = 2) per item, with the ranging of total score from 0 to 40 (see supplementary materials, S1 Table).

**Table 2:** The demographic characteristics of the participants.

	Phase 2 n = 223	Phase 3 n = 117
**Characteristic**	Mean (SD) or N (%)	Mean (SD) or N (%)
Age	71.42 (8.80)	72.71 (7.42)
Sex		
Male	53 (23.8)	28 (23.9)
**Education**		
Uneducated	26 (11.7)	15 (12.9)
Elementary school	70 (31.4)	37 (31.6)
Junior high school	25 (11.2)	10 (8.5)
Senior high school	50 (22.4)	26 (22.2)
Associate, bachelor’s degree and above	52 (23.3)	29 (24.8)
**Marriage**		
Single, divorced, widowed	73 (32.7)	44 (37.6)
Married, cohabited	150 (67.3)	73 (62.4)
**Living status**		
Living alone	40 (17.9)	26 (22.2)

**Table 3:** The items removed in Phase 2.

Item	Mean	SD	Proportion of the rating scale			Item-total correlation (Pearson’s r)	High vs. Low Group Comparison (Cohen’s d)	Cronbach’s α Change (if item deleted)
			0	1	2			
Two servings of foods high in cholesterol, sodium or saturated/trans fat	1.75	0.52	4.04	17.04	78.92	0.135	0.18 (n.s.)	0.002
Stay up late at night	1.39	0.80	19.73	21.08	59.20	0.142	0.16 (n.s.)	0.010
Not having enough rest	1.52	0.70	12.11	23.32	64.57	0.178	0.24 (n.s.)	0.005
Have enough rest in daytime	1.58	0.72	13.68	14.53	71.79	0.287**	0.45 (n.s.)	−0.002

**p < 0.01

### Phase 3: Evaluating the psychometric properties of HELP-T-SF

#### Descriptive data.

In this phase, we invited a subsample of 117 older adults from Phase 2 to complete the retest of the HELP-T-SF within 7–14 days after the initial assessment. The mean age of 72.71 (7.42) years (ranging from 55 to 89 years); 89 participants (76.1%) were women. The mean (standard deviation) of the total score for HELP-T-SF was 23.38 (6.78) (Table 4), with scores ranging from 9 to 40. No ceiling or floor effects were observed. During the data collection process, the reduction in the number of items to one-third of the original HELP-T facilitated significant time savings, taking 10–15 mins to finish.

**Table 4:** Psychometric properties of HELP-T-SF.

Item	Mean	SD	Proportion of the rating scale			Item-total correlation (Pearson’s r)	High vs. Low Group Comparison (Cohen’s d)	Cronbach’s α Change (if item deleted)
			0	1	2			
01. Walk for 20 min	1.24	0.81	23.08	29.91	47.01	0.485**	1.18**	−0.012
02. Do yoga or stretching exercise	1.15	0.80	25.64	34.19	40.17	0.535**	1.64**	−0.016
03. Do moderate intensity physical activity for 30 minutes	0.80	0.83	46.15	27.35	26.50	0.375**	0.98**	−0.004
04. Three servings of healthy foods rich in protein	1.51	0.68	10.26	28.21	61.54	0.492**	1.30**	−0.013
05. Three servings of fruits or vegetables	1.58	0.62	6.84	28.21	64.96	0.494**	1.14**	−0.013
06. Family caregiving, volunteering, or paid work	0.68	0.84	56.41	19.66	23.93	0.397**	1.57**	−0.005
07. Participate in social gatherings	1.50	0.74	14.53	20.51	64.96	0.499**	1.22**	−0.013
08. Gathering with family	0.75	0.82	48.72	27.35	23.93	0.382**	1.37**	−0.004
09. Contact with family members who don’t live together	1.23	0.77	20.51	35.90	43.59	0.475**	0.87**	−0.012
10. Engage in hobbies or interest activities at home	1.00	0.84	35.04	29.91	35.04	0.499**	1.40**	−0.013
11. Engage in outdoor hobbies or interest activities	0.63	0.75	52.99	30.77	16.24	0.322**	0.74**	−0.001
12. Engage in cognitive activities	1.17	0.80	24.79	33.33	41.88	0.530**	1.32**	−0.016
13. Have good quality of sleep	1.42	0.76	16.24	25.64	58.12	0.303**	0.63*	0.000
14. Do family chores	1.29	0.81	22.22	26.50	51.28	0.484**	1.16**	−0.012
15. Have a sense of satisfaction in life	1.62	0.58	5.13	27.35	67.52	0.424**	1.53**	−0.009
16. Do things that bring good moods	1.39	0.73	14.53	31.62	53.85	0.443**	1.48**	−0.010
17. Do relax activities	1.09	0.83	29.91	30.77	39.32	0.516**	1.69**	−0.014
18. Read health-related articles	0.71	0.75	47.01	35.04	17.95	0.429**	0.95**	−0.008
19. Watch health-related programs	1.20	0.79	23.08	34.19	42.74	0.388**	0.89**	−0.005
20. Monitor health at home	1.40	0.80	19.66	20.51	59.83	0.366**	0.85**	−0.004

**p < 0.01 *p < 0.05

#### Reliability.

The Cronbach’s α for the total score of the HELP-T-SF was 0.781 (95% CI: 0.54 to 0.89), indicating acceptable internal consistency. Additionally, the test-retest reliability of the HELP-T-SF total scores, measured by the ICC with a 95% confidence interval (CI), was 0.782 (95% CI: 0.54 to 0.89), demonstrating acceptable score agreement (Table 4).

#### Validity.

All items demonstrated significant correlations with the total score, ranging from 0.303 to 0.535 (Table 3), thereby supporting the construct validity of the HELP-T-SF. The concurrent validity between the total score of HELP-T-SF and the original HELP-T was evidenced by a significant correlation of 0.749 (p < 0.001, 95% CI: 0.56 to 0.86), indicating a moderate to high correlation (r = 0.6 or above). Additionally, convergent validity was assessed, revealing significant weak to low positive correlations with well-being (0.311), self-perceived quality of life (0.319), self-perceived health (0.245), and health satisfaction (0.211), all at p < 0.001, showing they are different concepts.

## Discussions

This study developed and evaluated the reliability and validity of the HELP-T-SF. Participants completed the items in 10–15 minutes, reducing the time required for the full HELP-T by half. Internal consistency and test-retest reliability indicated acceptable reliability. A strong correlation between the HELP-T-SF and the original HELP-T supports the construct validity of the short form in capturing lifestyle activity engagement among older adults. Grounded in the OTPF framework, it evaluates lifestyle profiles through activity engagement, specifically for the older adults. The HELP-T-SF facilitates a holistic, client-centered approach to identifying lifestyle changes and complements LM principles from a medical perspective.

For general population, lifestyle assessments increasingly being developed, including tools such as the Lifestyle Medicine Health Behavior Scale (LMHB) [37], the Lifestyle Assessment Long and Short Forms, and the Lifestyle Medicine Assessment Tool. Among those assessments, the HELP-T-SF shares similarities with the LMHB (evaluated using the six-pillar framework [12]). However, it does not include a leisure domain related to lifestyle activity engagement, which is particularly important for the older adults.

Several countries have developed older adults lifestyle assessments in the past decade, yet no consensus exists on a definitive framework. Examples include the Fantastic Life Inventory (FLI, from Poland) [38], the Questionnaire on Health-Promoting Lifestyles of the Elderly (from China) [39], the Elderly Lifestyle Profile (from South Korea) [40], the Healthy Lifestyle Questionnaire for the Elderly (HEAL, from Iran) [41], the Individual Lifestyle Profile Scale (ILP, from Portugal) [42], and the Healthy Lifestyle Profile Scale for Elderly (HLPSE, from Sri Lanka) [43]. These assessments vary in item count (15–62), domain classification (3–9 domains), and scoring methods (frequency based, Yes/No, engagement levels, mixed methods). All assessments include physical activities, and four out of six studies address nutrition. The rest domains are social, mental, risk and health behavior, ADL and others (see supplementary file in details, S2 Table).

To outline a healthy lifestyle profile in a concrete and measurable manner, the HELP-T-SF was designed with a structured framework, emphasizing of actual participation in health-promoting activities in daily life, rather than merely assessing knowledge of related concepts or the presence of unhealthy conditions. It aims to provide a practical tool from a behavior change perspective.

To ensure consistency with the original HELP-T, items were carefully reviewed, selected, combined, or rewritten while preserving the seven-domain structure with two to four items per domain. Reverse items, included in the original HELP-T, were excluded in the HELP-T-SF due to their potential to compromise psychometric properties. In measurement and evaluation research, the inclusion of reverse items primarily poses a risk to the precision of the instrument’s measurements and may jeopardize the interpretation of its unidimensionality. Consequently, the variance in the combined form may be diminished, resulting in significantly poorer psychometric properties [44]. Additionally, the HELP-T employs frequency-based scoring within a Likert scale, and reverse scoring may result in lead to inconsistent score intervals. For example, “4-7 days/week” and “none or 1-3 days/month,” may not be equal due to reverse scoring, which diminishes clinical relevance.

Our findings indicate a moderate correlation between HELP-T-SF and quality of life and well-being, with a lower correlation with health. This suggests that although lifestyle is related to both health and quality of life, it remains a distinct concept, warranting further exploration in future studies.

Recognizing sleep as a crucial lifestyle component, we retained one positively phrased sleep-related item in the final HELP-T-SF. Sleep significantly influences lifestyle by affecting other health-related behaviors, including dietary habits, physical activity, and mental health [45]. Multicomponent Lifestyle Medicine interventions have demonstrated effectiveness in improving sleep quality among those with significant sleep disturbances [46]. Recognizing sleep as an essential component of lifestyle, we added two positively phrased items to the preliminary version of HELP-T-SF, and retained one in the ADL domain of the final version.

We acknowledge the 10-year gap between Phase 1 (2012–2013) and Phases 2–3 (2023–2024). However, the core behaviors measured by the HELP-T-SF—such as physical activity, social participation, and healthy routines—are relatively stable over time. In Phase 2, we carefully reviewed item relevance and excluded outdated content. For instance, while older adults previously listened to radio broadcasts via traditional devices, many now use smartphones for similar activities. This illustrates changes in medium rather than behavior, and such adjustments were considered during item selection to ensure current applicability.

The participant demographics revealed some limitations. The sample consisted primarily of women, with most having only primary education. This bias reflects our data collection from community care stations in Taiwan, where older adults with lower education levels are more prevalent. However, if these individuals were able to effectively use the HELP-T-SF, it is unlikely that those with higher education would encounter difficulties. Further research is needed to validate these findings.

## Conclusions

The HELP-T-SF, developed through a three-phase process from the HELP-T, is a simpler and more efficient tool for assessing the lifestyle profile of older adults. It demonstrates acceptable reliability and validity based on CTT analyses.

This study provides a quicker, validated instrument for assessing the lifestyle profiles of community-dwelling older adults in Mandarin-speaking populations. By evaluating lifestyle through activity arrangement and engagement, the HELP-T-SF offers healthcare practitioners in Lifestyle Medicine a practical tool to establish client-centered goals and design personalized interventions effectively.

## Supporting information

S1 Table The final version of Health Enhancement Lifestyle Profile – Taiwanese Short Form Version (HELP-T-SF). (PDF)

S2 Table Comparison of elderly lifestyle assessments during past decade. (PDF)

S3 Dataset Minimal dataset. (ZIP)
