# Supplementary material for: Development of Health Enhancement Lifestyle Profile - Taiwanese Short Form Version (HELP-T-SF) for the community-dwelling elderly
Source: PLoS One. 2025 Nov 12;20(11):e0336695. doi: 10.1371/journal.pone.0336695 (PMC12611165; doi:10.1371/journal.pone.0336695)
Supplement: S1 Table — (PDF) [file pone.0336695.s001.pdf]

S1 Table. The final version of Health Enhancement Lifestyle Profile - Taiwanese Short Form Version (HELP-T-SF)

Please check according to your actual lifestyle conditions over the past one month.<br>How often during a week do you…?	None or 1-3 days/month	1-3 days/week	4-7 days/week
1. walk outside or on a treadmill for at least 20 minutes at a time.	☐	☐	☐
2. perform stretching or flexibility exercises (such as joint mobility/stretching, calisthenics, towel exercises, or yoga).	☐	☐	☐
3. participate in moderate physical activities (such as cycling, jogging, mountain climbing, or hiking) for at least 30 minutes at a time.	☐	☐	☐
4. eat 3 or more servings of healthy foods rich in protein in one day (such as poultry, fish, beans, nuts, skim milk, cheese, tofu, or soy milk; one serving is approximately a palm or a cup).	☐	☐	☐
5. eat 3 or more servings of fruits and vegetables in one day (one serving is roughly half a bowl or the size of a fist for cooked food).	☐	☐	☐
6. help take care of grandchildren or family members; being volunteer, or engage in paid work.	☐	☐	☐
7. participate in social gatherings (such as religious activities, afternoon tea, community courses, karaoke, playing chess, hiking).	☐	☐	☐
8. gather with relatives and friends (who do not live together).	☐	☐	☐
9. meet with family members or interact through communication apps (including those who do not live together).	☐	☐	☐
10. engage in hobbies or activities at home that interest you (such as reading news or magazines, calligraphy, painting, caring for pets, gardening, take part in carpentry, auto/house fixing).	☐	☐	☐
11. participate in hobbies or activities you enjoy outside the home (such as shopping at markets, traveling, connecting with nature, fishing, visiting hot springs, or attending museums or exhibitions).	☐	☐	☐

Please check according to your actual lifestyle conditions over the past one month.<br>How often during a week do you…?	None or 1-3 days/month	1-3 days/week	4-7 days/week
12. engage in mental activities (such as playing chess or cards, writing diaries, artistic creation, financial investing, crafts activities or various learning courses).	☐	☐	☐
13. sleep well (such as fall asleep easily, have deep sleep, wake up and fall back asleep).	☐	☐	☐
14. handle household chores (such as preparing meals, shopping for household essentials, making deposits, paying bills).	☐	☐	☐
15. feel a sense of happiness and satisfaction in life due to the things you do throughout the day.	☐	☐	☐
16. spend at least 20 minutes in a day doing simple things that can bring about your good moods (such as caring for pets, or singing, reading, listening to music etc.)	☐	☐	☐
17. engage in relaxation activities to unwind (such as meditation, yoga, listening to music, massages, spa treatments, soaking in hot springs, or other activities)	☐	☐	☐
18. read health-related articles, editorials, materials, or magazines	☐	☐	☐
19. watch or listen to health-related programs on TV, radio, or online	☐	☐	☐
20. manage your health at home (such as taking medications on time and in appropriate amounts, measuring blood pressure, heart rate, blood sugar levels, or weight)	☐	☐	☐

健康生活型態剖析量表—台灣簡短版（2025 版）

**請依照過去一個月以來之實際生活型態狀況進行勾選，回答您一星期有幾天從事以下活動？**	無 或<br>1~2 天/月	1~3<br>天/週	4~7<br>天/週
1. 出外散步或使用跑步機走路作為運動一次至少 20 分鐘	☐	☐	☐
2. 做伸展或柔軟操運動（例如關節活動/伸展運動、健身操、毛巾操或瑜伽）？	☐	☐	☐
3. 中度身體活動（例如腳踏車、慢跑、爬山或健行）一次至少 30 分鐘	☐	☐	☐
4. 吃三份或以上富含蛋白質的健康食物（例如家禽、魚類、豆類、堅果類、脫脂牛奶、乾酪、豆腐或豆漿。一份約為一掌心/一杯）	☐	☐	☐
5. 吃三份或以上蔬菜和水果？（一份大約為半碗或一個拳頭大小之熟食）	☐	☐	☐
6. 幫忙照顧孫子或其家人或擔任志工或從事有薪工作	☐	☐	☐
7. 參加與人互動的聚會（例如：宗教活動、下午茶、社區課程、卡拉 ok、下棋、爬山）	☐	☐	☐
8. 與親朋好友（不住一起的）聚會？	☐	☐	☐
9. 與家人見面或用通訊軟體互動（含不住在一起的家人）	☐	☐	☐
10. 在家從事您覺得有興趣的嗜好或活動（例如：閱讀、寫作、下棋、拼圖、書法、繪畫、寵物、園藝）	☐	☐	☐

**請依照過去一個月以來之實際生活型態狀況進行勾選，回答您一星期有幾天從事以下活動？**	無 或 1~2 天/月	1~3 天/週	4~7 天/週
11. 出門從事您覺得有興趣的嗜好或活動（例如：外出逛街/逛市場、出遊/接近大自然/釣魚/泡溫泉、博物館或展覽）	☐	☐	☐
12. 從事動腦活動（例如：下棋打牌等益智遊戲、閱讀書報雜誌、藝術創作、園藝、投資理財、各種學習課程等）	☐	☐	☐
13. 睡眠品質良好？（例如：容易入睡、不淺眠、醒來可以再睡回去）	☐	☐	☐
14. 做家庭事務（例如：準備餐點、採買家中生活必需品、提存款、繳帳單）？	☐	☐	☐
15. 因為您一天中所做的事情而感到快樂或滿足？	☐	☐	☐
16. 花至少 20 分鐘做簡單而能給您好心情的事情（例如照顧寵物、唱歌、閱讀、聽音樂…等）？	☐	☐	☐
17. 做放鬆活動以放鬆自己（例如冥想、做瑜珈、聽音樂、按摩、做 SPA、泡湯或其他）？	☐	☐	☐
18. 閱讀健康相關文章、社論、資料或雜誌？	☐	☐	☐
19. 收看或收聽健康相關電視、廣播或網路節目？	☐	☐	☐
20. 您一星期有幾天會在家做健康管理（例如：按時按量服藥、測量血壓、心跳、血糖指數或體重）？	☐	☐	☐
