# Supplementary material for: Development of Health Enhancement Lifestyle Profile - Taiwanese Short Form Version (HELP-T-SF) for the community-dwelling elderly
Source: PLoS One. 2025 Nov 12;20(11):e0336695. doi: 10.1371/journal.pone.0336695 (PMC12611165; doi:10.1371/journal.pone.0336695)
Supplement: S2 Table — (PDF) [file pone.0336695.s002.pdf]

S2 Table. Comparison of elderly lifestyle assessments during past decade

name of the assessment	country	(author, years)	number of items	number of domains	name of the domains/number of items in each domain	physical activity	nutrition	social/productive	mental/spiritual	risk/health behavior	ADL	Leisure	others
FANTASTIC Life Inventory	Poland	(Deluga et al., 2018)	25	9	F – family and friends / 2<br>A – physical activity / 2<br>N – nutrition / 3<br>T – tobacco toxics / 4<br>A – alcohol / 3<br>S – sleep, seatbelt, stress, safe sex / 5<br>T – type of personality / 2<br>I – insight / 3<br>C – career / 1	physical activity	nutrition	family and friends	stress	tobacco<br>alcohol	sleep	-	seatbelt, safe sex<br>type of personality<br>insight<br>career
Questionnaire on Health-Promoting Lifestyles of the Elderly	China	(Changliang Du, 2019)	50	3	Physical health / 26<br>Mental health / 13<br>Social well being / 11	physical health - physical condition, exercise habits	physical health - eating routine	social well being - prosocial behaviors, interpersonal relationships (including family), social recognition	mental health - mental status, emotion control, stress handling, self-regulation	physical health - use of drug or alcohol	physical health - quality of sleep	-	physical health - living environment
the elderly lifestyle profile	South Korea	(Park & Park, 2020)	62	3	physical activity / 18<br>activity participation / 20<br>nutrition / 24	physical activity	nutrition	activity participation-social activity/productive activity	-	nutrition-smoking/alcohol	activity participation-routine/ADL/ rest and sleep	activity participation - Leisure activity	-

The healthy lifestyle questionnaire for elderly (heal)	Iran	(Bandari et al., 2020)	35	8	**persopnal health and hygiene / 2**<br>**performing life tasks independently / 3**<br>**sport / 2**<br>**nutrition / 8**<br>**mental health, sleep and rest / 5**<br>**observing safety and health advice / 8**<br>**social and family relations / 2**<br>**spritual and religious activities / 5**	**sport**	**nutrition**	**social and family relation**	**mental health, sleep and rest spritual and religious activities**	**observing safety and health advice**	**persopnal health and hygiene performing life tasks independently**	**-**	**-**
Individual Lifestyle Profile Scale (ILP)	Portugal	(Alves Faria et al., 2022)	15	4	**health self-management dimension / 6**<br>**social participation and group interaction dimension / 4**<br>**citizenship dimension / 3**<br>**physical activity dimension / 2**	**physical activity dimension**	**health self-management dimension - about nutrition**	**social participation and group interaction dimension 4**	**-**	**health self-management dimension - about smoke/alcohol**	**-**	**-**	**citizenship dimension**
Healthy Lifestyle Profile Scale for Elderly (HLPSE)	Sri Lanka	(Jayasinghe et al., 2024)	28	8	**physical activity / 5**<br>**mental health / 5**<br>**nutritional intake / 4**<br>**dietary concerns / 4**<br>**social relationships / 3**<br>**risk behaviors / 3**<br>**health responsibilities / 2**<br>**body consciousness / 2**	**physical activity**	**nutritional intake dietary concerns**	**social relationships**	**mental health**	**risk behaviors**	**-**	**-**	**health responsibilities body consciousness**
